# Influence of technical and maternal-infant factors on the measurement and expression of extracellular miRNA in human milk

**DOI:** 10.3389/fimmu.2023.1151870

**Published:** 2023-07-10

**Authors:** Elizabeth A. Holzhausen, Allison Kupsco, Bridget N. Chalifour, William B. Patterson, Kelsey A. Schmidt, Pari Mokhtari, Andrea A. Baccarelli, Michael I. Goran, Tanya L. Alderete

**Affiliations:** ^1^ Department of Integrative Physiology, University of Colorado Boulder, Boulder, CO, United States; ^2^ Department of Environmental Health Sciences, Columbia University Mailman School of Public Health, New York, NY, United States; ^3^ Department of Pediatrics, Children’s Hospital Los Angeles, Los Angeles, CA, United States

**Keywords:** EV-miRNA, breast milk, human milk, microRNA, miRNA

## Abstract

Breast milk contains thousands of bioactive compounds including extracellular vesicle microRNAs (EV-miRNAs), which may regulate pathways such as infant immune system development and metabolism. We examined the associations between the expression of EV-miRNAs and laboratory variables (i.e., batch effects, sample characteristics), sequencing quality indicators, and maternal-infant characteristics. The study included 109 Latino mother-infant dyads from the Southern California Mother’s Milk Study. Mothers were age 28.0 ± 5.6 and 23-46 days postpartum. We used principal components analysis to evaluate whether EV-miRNA expression was associated with factors of interest. Then, we used linear models to estimate relationships between these factors and specific EV-miRNA counts and analyzed functional pathways associated with those EV-miRNAs. Finally, we explored which maternal-infant characteristics predicted sequencing quality indicators. Sequencing quality indicators, predominant breastfeeding, and breastfeedings/day were associated with EV-miRNA principal components. Maternal body mass index and breast milk collection timing predicted proportion of unmapped reads. Expression of 2 EV-miRNAs were associated with days postpartum, 23 EV-miRNAs were associated with breast milk collection time, 23 EV-miRNAs were associated with predominant breastfeeding, and 38 EV-miRNAs were associated with breastfeedings/day. These EV-miRNAs were associated with pathways including Hippo signaling pathway and ECM-receptor interaction, among others. This study identifies several important factors that may contribute to breast milk EV-miRNA expression. Future studies should consider these findings in the design and analysis of breast milk miRNA research.

## 1 Introduction

Breast milk has significant health benefits and promotes infant growth (1) and immune development (2). For example, breastfed infants have lower risk of gastrointestinal (3) and respiratory infection (4), childhood obesity (5), asthma and allergy (6), and childhood morbidity and mortality (7). In addition to containing ideal infant nutrition, breast milk is also a rich source of thousands of bioactive compounds, including extracellular vesicles (EVs). EVs are nano-sized membrane vesicles actively released by breast epithelial cells and other cell types (8). These EVs contain microRNA (miRNA) and emerging evidence indicates that these may be part of a signaling system that contributes to infant growth and development (9, 10).

miRNAs are non-coding RNAs that regulate post-transcriptional gene expression by targeting mRNAs for degradation or repression. Breast milk miRNAs can be contained within EVs, conferring resistance to harsh environments. They can survive *in vitro* digestion that mimics the infant gut (11–14), where they may be taken up by infant epithelial cells (13, 14) and can cause *in vitro* changes to gene expression (12). Although still controversial, some posit that human milk EV-miRNAs produced by the mother may regulate infants’ gut epithelial interface and potentially impact postnatal gut maturation, nutrient uptake, and infant health through the local regulation of post-transcriptional gene expression (10).

Despite the emergence of breast milk EV-miRNAs as a potentially important factor in infant growth and development, the technical and biological factors that impact their expression have not been fully characterized in a population-based cohort. Some previous studies investigating EV-miRNA expression have found that EV-miRNA expression is associated with days postpartum, maternal BMI, and smoking (15). Another study found differences in exosomal miRNA expression of miR-148a and miR-30b in mothers with overweight and obesity compared to normal weight mothers at 1-month postpartum. Expression of these miRNAs were also associated with infant growth and body composition (16). Exosomal miRNA expression also appears to differ based on both type 1 (17) and gestational (18) diabetes. While these previous studies have laid the foundation for understanding the biological factors associated with breast milk miRNA content, few have focused on EV-miRNAs or reported on technical variables including laboratory variables and sequencing quality indicators.

The primary aim of the current study was to determine which technical and biological variables should be taken into consideration in the design and analysis of future breast milk EV-miRNA research. Specifically, we examined the relationships between breast milk EV-miRNAs with laboratory variables (i.e., batch effects, sample characteristics), sequencing quality indicators, and maternal and infant characteristics. As a secondary aim, we sought to determine if maternal and infant characteristics predicted sequencing quality indicators, including the proportion of unmapped reads and proportion of ribosomal RNA. Results from this study can be used to inform future investigations of breast milk EV-miRNAs.

## 2 Materials and methods

### 2.1 Study participants

The Southern California Mother’s Milk Study is an ongoing, longitudinal cohort of Latino mother-infant dyads recruited from Los Angeles County maternity clinics from 2016- 2019, which has been previously described (19). The primary aim of the Mother’s Milk Study was to assess the impact of sugars and human milk oligosaccharides on the infant microbiome and obesity. Inclusion criteria were: ≥18 years at time of delivery; healthy, singleton birth; enrollment by 1-month postpartum; and the ability to read at the 5^th^ grade level in Spanish or English. Individuals were excluded if they reported diagnoses or medications known to affect physical/mental health, nutritional status, or metabolism; were current tobacco or recreational drug users; had pre-term or low birth weight infants; or had infants with clinically diagnosed fetal abnormalities. The Institutional Review Boards of the University of Southern California, Children’s Hospital of Los Angeles, and the University of Colorado Boulder approved the study procedures. Written informed consent was obtained from participants prior to study enrollment.

### 2.2 Study design

Visits occurred at 1-, 6-, 12-, 18-, and 24-months postpartum. 219 mother-infant dyads were enrolled, and 209 contributed breast milk samples at the 1-month visit. 111 samples from participants that had completed their 24-month visit were included for EV-miRNA sequencing. One sample failed sequencing. There were some differences (mother age and breastfeedings/day) between participants who contributed breast milk but were not included in EV-miRNA analysis and participants for whom EV-miRNA data was obtained (**Supplemental Table 1**). One observation was excluded from this analysis because their 1-month visit occurred later than the rest, at 62 days postpartum, resulting in a final analytic sample of 109.

Socioeconomic status (SES) was estimated using a modified version of the Hollingshead index (20), which has been previously described (19). Infants were categorized as early (<38 weeks gestation), on time (38-42 weeks gestation), and late (>42 weeks gestation) via maternal self-report. Visits between October 1-March 31 were categorized as cold season, while all other visits were considered warm season. Questionnaires were used to determine whether infants were predominantly breastfed (i.e., fed formula less than once/week) and breastfeedings/day. Breastfeedings/day was measured semi-quantitatively (participants selected from the following choices: 0-1, 1, 2, 3, 4, 5, 6, 7, and ≥ 8 feedings/day) but was treated as a continuous variable.

### 2.3 Breast milk collection

Breast milk was collected between 7AM-3PM, ≥1.5 hours after the previous feeding, and after the mother had fasted for ≥1 hour. Participants provided a single full expression from the right breast using an electric breast pump as previously described (21). Milk was frozen at -80°C until analysis.

### 2.4 miRNA sequencing, processing, and expression

EVs were isolated from stored samples as previously described (15). Briefly, samples were centrifuged to remove the lipid layer, then again to remove cellular debris and apoptotic bodies. EVs were extracted using the ExoEasy Maxi KIT (Qiagen, Germantown, MD) and total RNA was isolated with the miRNeasy Serum/Plasma Maxi KIT (Qiagen, Germantown, MD). Samples were cleaned using the RNA Clean & Concentrator-5 Kit (Zymo Research, Irvine, CA) and sample purity and quantity were measured on an Implen NanoPhotometer spectrophotometer (München, Germany).

Sequencing and library preparation was performed at the University of California San Diego. The NEBNext Small RNA Library Prep Set for Illumina (NEB, Ipswich, MA) was used to construct sequencing libraries with minor optimizations to the manufacturer’s protocol to account for low input and cell-free RNA. Reactions were conducted at one-fifth the recommended volume, adapters were diluted 1:6, and library amplification PCR used 17 cycles. Libraries were cleaned with the DNA Clean and Concentrator Kit (Zymo Research, Irvine, CA) and the concentrations were quantified using the Quant-iT PicoGreen dsDNA Assay (Invitrogen, Waltham, MA). Samples were pooled with equal volumes. The pools size distribution was determined with a DNA HS Chip on a BioAnalyzer (Agilent Technologies, Santa Clara, CA) before size selection (115-150 base pairs [bp]) on a Pippin Prep instrument (Sage, Beverly, MA) to remove adapter dimers and fragments larger than the target miRNA population. Libraries were sequenced to ~1,000,000 total reads per pool using a MiSeq instrument with a Nano flow cell (Illumina Inc, San Diego, CA). This sequencing data was used to balance the samples into new pools for deeper sequencing on a HiSeq4000 instrument using single-end 75 bp runs.

Sequencing data were mapped using the ExceRpt small RNA sequencing data analysis pipeline on the Genboree Workbench (http://genboree.org/site/exrna_toolset/) (22). Samples were mapped using default parameters, except for filtering to a minimum read length of 15 nucleotides with zero mismatches. Quality control was performed according to External RNA Controls Consortium (ERCC) guidelines (22). One sample had less than 100,000 transcriptome reads and was removed from the analysis. Raw EV-miRNA read counts were normalized using the trimmed mean of M (TMM) method from the EdgeR package (23) and were converted into the z-score of counts per million (CPM) for principal components analysis.

### 2.5 Extracellular vesicle characterization

To characterize the isolated EVs, we evaluated the concentration, sizes, and distribution of four randomly selected human milk EV samples using nanoparticle tracking analysis on the ViewSizer 3000 (Horiba Scientific, Piscataway, NJ) as previously described (24), with the blue laser set to 210 mW, the green laser set to 12 mW, and the red laser set to 8 mW. Samples were diluted 1:3000 in sterile PBS and normalized to a blank PBS sample. The camera’s frame rate was 30 frames per second for 15 ms exposure with a gain of 30. We recorded 60, 30-second videos with 300 frames per video.

To verify the presence and purity of the isolated EVs, we used the Exo-Check Exosome Antibody Assay (System Biosciences, Palo Alto, CA) to measure the levels of eight EV indicators and a cellular control according to manufacturer instructions. This was performed on four sets of three pooled EV samples and three sets of three matched EV-depleted samples, where EV-depleted samples were the remaining flow through following EV affinity captures from the milk, which would be considered EV-depleted (EV-depleted milk was not available for the fourth set of pooled samples). Protein was quantified using a BCA assay and blots were imaged with the Azure 400 Visible Fluorescent Western Blot Imaging System (Azure Biosystems, Dublin, CA). Five randomly selected human milk EV samples also underwent transmission electron microscopy at 50,000x and 100,000x magnification at the Weill Cornell Medical College Electron Microscopy & Histology Core to further confirm the purity of our EV preparation and to characterize subpopulations of EVs in our samples. All relevant EV characterization data have been submitted to the EV-TRACK knowledgebase (EV-TRACK ID: EV220416), where our EV-metric was 50% (25).

### 2.6 Laboratory variables and sequencing quality indicators

Laboratory variables and indicators of sequencing quality (22) were considered potential covariates. Date of EV extraction was analyzed as a proxy for batch effects related to EV isolation. Milk samples, initially 0.5 mL, were centrifuged twice to remove the lipid layer, cellular debris, and apoptotic bodies. The remaining volume was the skim milk (mean: 170.45 ± 87.21μL). The proportion of ribosomal RNA (rRNA) reads was the proportion of total input reads which were rRNA (mean: 0.12 ± 0.05). In cell-free RNA samples, a high proportion of rRNA reads can indicate contamination by cellular material. The proportion of unmapped reads was the proportion of total input reads that could not be mapped to the human reference genome given our alignment criteria (mean: 0.32 ± 0.11). A high proportion of unmapped reads can indicate the presence of RNA editing or contamination by exogenous genomes. The transcriptome to genome ratio (transcriptome:genome ratio) was the transcriptome reads divided by the genome reads. A low transcriptome:genome ratio (<0.5 as suggested by previous studies (22)) may indicate contamination with cellular RNA. Nine samples with low transcriptome:genome ratios were included in the main analyses, but excluded as a sensitivity analysis (**Supplemental Table 2**).

### 2.7 Statistical analysis

Two approaches were used to determine which factors were most strongly associated with breast milk EV-miRNAs: principal components analysis (PC) and linear regression. PC analysis is a data reduction technique which allowed us to summarize the variability in high-dimensional miRNA data using a few principal components, which we then interpret as EV-miRNA “profiles”, or a general characterization of EV-miRNA expression. Linear regression analysis allowed us to examine the relationship between individual EV-miRNAs and characteristics of interest.

Briefly, we examined whether the proportion of rRNA reads, volume of skim milk, date of EV extraction, low transcriptome:genome ratio, or proportion of unmapped reads were associated with EV-miRNA profile. Using the prcomp package (26) in R, we focused on PCs 1 and 2, which cumulatively explained 34.4% of variability in EV-miRNA expression (**Supplemental Figure 1**). We found that volume of skim milk, proportion of rRNA reads, proportion of unmapped reads, and low transcriptome:genome ratios were associated with PC 1 and/or 2. We also examined whether maternal-infant characteristics were associated with EV-miRNAs, adjusting for proportion of rRNA and volume of skim milk. In our main analysis, we only adjusted for proportion of rRNA since all sequencing quality indicators (e.g., proportion of rRNA, proportion of unmapped reads, and low transcriptome:genome ratio) were highly correlated (r range: -0.38, 0.50; p_all_<0.004). As a sensitivity analysis, we adjusted for the proportion of unmapped reads instead of the proportion of rRNA. Unadjusted p-values are reported, as are p-values after adjustment for false discovery rate (FDR), using the Benjamini-Hochberg procedure (27), with a threshold of P_FDR_<0.10 for statistical significance. Following this, we used linear models to estimate the associations between log-transformed EV-miRNA counts per million and maternal-infant characteristics. These analyses adjusted for proportion of rRNA and volume of skim milk. As a sensitivity analysis, we adjusted for proportion of unmapped reads instead of proportion of rRNA.

**Figure 1:**
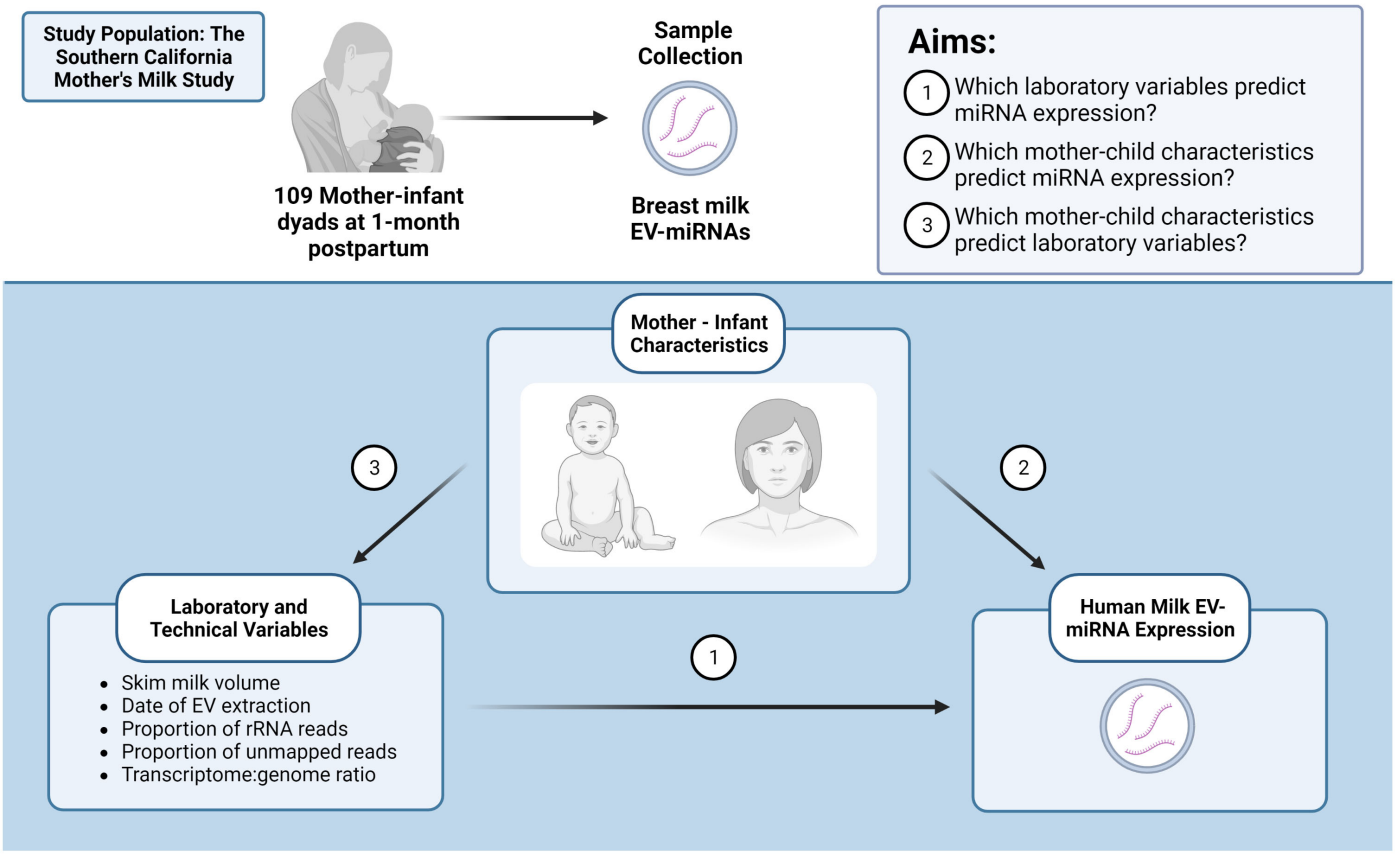
Identifying important factors that may contribute to human milk EV-miRNA expression. Created with BioRender.com.

Lastly, given that the proportion of unmapped reads and rRNA vary across samples, we sought to determine whether mother-infant characteristics predicted these sequencing quality indicators using linear regression analysis. Because maternal BMI and breast milk collection time predicted proportion of unmapped reads, we re-ran our linear models without adjusting for sequencing quality indicators. Specifically, we re-examined the associations between BMI and breast milk collection time with EV-miRNA expression. This additional analysis was performed since differential results with and without adjustment for sequencing quality indicators would suggest that the proportion of unmapped reads or rRNA may mediate the relationship between BMI or breast milk collection time with EV-miRNA expression (**Supplemental Figure 2**). For a visual description of study aims and relationships examined, see **Figure 1** (Created with BioRender.com).

### 2.8 miRNA pathway and ontology analysis

EV-miRNAs associated with maternal-infant characteristics underwent functional annotation with DIANA MirPATH version 3 online software (https://dianalab.e-ce.uth.gr/html/mirpathv3/index.php?r=mirpath) (28). Reads aligned to precursor EV-miRNAs (n = 3) were input as their mature counterparts for pathway and ontology analyses. We utilized microT-CDS [v5.0] (*in silico* prediction of miRNA-gene interactions (29)) and TarBase v7.0. (a catalogue of experimentally validated miRNA-gene interactions (30)) to predict mRNA targets. We then used MirPATH to identify Kyoto Encyclopedia of Genes and Genomes (KEGG) pathways (31) with significant enrichment (P_FDR_ ≤ 0.05).

## 3 Results

### 3.1 Study population characteristics

General study population characteristics are shown in **Table 1**. Briefly, breast milk was collected at the 1-month visit, which was an average of 32.5 days postpartum (range: 23-46 days). Mothers were 28.0 ± 5.6 years old and had a mean BMI of 29.9 ± 4.7 kg/m^2^, with 16% of participants having normal weight, 39% having overweight. (25 ≤ BMI < 30 kg/m^2^), and 45% having obesity (BMI ≥ 30 kg/m^2^).

**Table 1:** Characteristics of mother-infant dyads from the Southern California Mother’s Milk study at 1-month of infant age.

Maternal and infant characteristics	Mean ± SD or N, %
Maternal age (years)	28.0 ± 5.6
Socioeconomic status	25.6 ± 12.2
Pre-pregnancy BMI (kg/m^2^)	28.3 ± 5.4
Maternal BMI (kg/m^2^)	29.9 ± 4.7
Normal weight (BMI < 25)	17, 16%
Overweight (25 ≤ BMI < 30)	43, 39%
Obese (BMI ≥ 30)	49, 45%
Infant sex (female, male, % female)	60, 49, 55%
Mode of delivery (vaginal, caesarean, % vaginal)	83, 26, 76%
Days postpartum	32.5 ± 3.3
Gestational age	
Early (38-40 weeks gestation)	27, 25%
On time (40 weeks gestation)	55, 50%
Late (40-42 weeks gestation)	27, 25%
Gestational diabetes (Yes, No, %Yes)	7, 102, 6.4%
**Breast milk collection timing**	Mean ± SD or N, %
Breast milk collection time (hours past midnight)	11.3 ± 1.5
Season of collection (cold, warm, % cold)	59, 50, 54%
**Infant feeding characteristics**	Mean ± SD or N, %
Predominantly breastfed (yes, no, % yes)	48, 61, 44%
Breastfeedings per day	6.4 ± 2.4

Data are reported mean and standard deviation (SD) unless otherwise noted.

### 3.2 Characterization of human milk EVs

**Figure 2:**
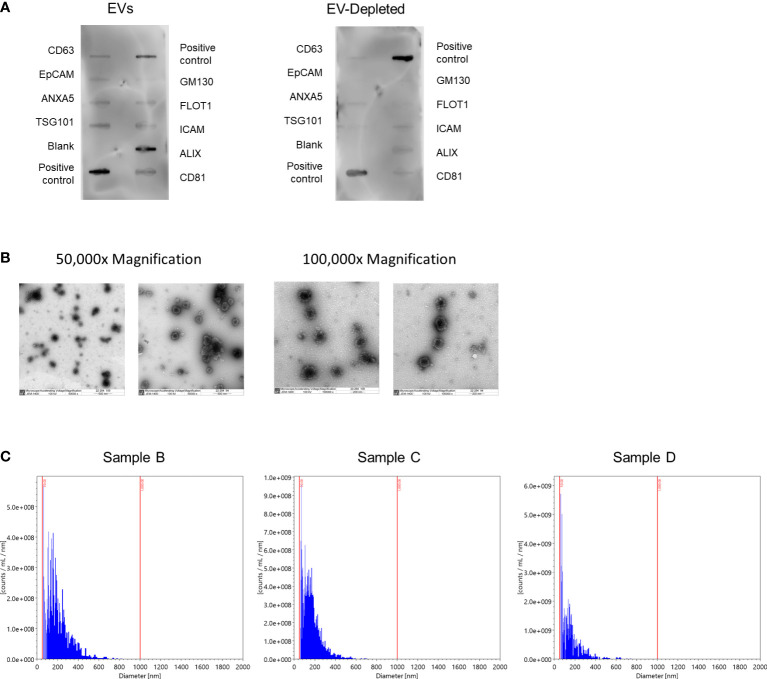
Characterization of human milk EVs in representative samples. **(A)** Representative results from Exo-Check Array demonstrating exosome-related protein expression in a pooled sample of three human milk EV samples (left), and the corresponding EV-depleted samples (right), where the darkness of each line indicates the presence of the indicated protein. GM130: Cis-golgi matrix protein, FLOT1: Flotillin-1, ICAM1: Intracellular adhesion molecule 1, ALIX: Programmed cell death 6 interacting protein (PDCD6IP), CD81: Tetraspanin, CD63: Tetraspanin, EpCam: Epithelial cell adhesion molecule, ANXA5: Annexin A5, TSG101: Tumor susceptibility gene 101. **(B)** Representative transmission electron microscopy images at 50,000x (left) and 100,000x magnification. EVs are the larger dots (electron rich areas), surrounded by lighter membranes. **(C)** Histograms for the number of particles per milliliter of EVs by particle diameter with bins logarithmically scaled.

To confirm the presence of EVs in our sample preparation, we characterized EVs in four sets of three pooled randomly selected samples (**Table 2**). The average EV concentration was 6.9x10^14^ particles/mL of skim milk (e.g., following centrifuge). The mean EV size was 184.5 nm ± 105.3. Next, we semi-quantitatively measured eight known exosome markers and a marker of cellular contamination (**Figure 2A**; **Supplemental Figure 3**), visualized exosomes using transmission electron microscopy (TEM) (**Figure 2B**), and measured EV diameters using nanoparticle tracking analysis (**Figure 2C**). **Figure 2A** shows a representative image of array results. All results can be found in **Supplemental Figure 3**. While array results indicate a small amount of cellular contamination (GM130), EV samples were positive for EV markers CD63, EpCAM, ANXA5, TSG101, FLOT1, ICAM, ALIX, and CD81 and had higher levels of all positive markers in comparison to their paired EV-depleted samples. The mean protein concentration in the EV samples was 0.64 µg/µl of skim milk ± 0.23, and the mean protein concentration in EV-depleted samples was 2.7 µg/µl of skim milk ± 1.0. TEM images at 50,000x and 100,000x magnification confirm the presence of EVs of the expected size, and nanoparticle tracking analysis indicated that EVs were of the size range expected and there were no large contaminating particles.

**Table 2:** Characterization of extracellular vesicles in four randomly selected human milk samples.

Sample	Concentration (particles/mL skim milk)	Average EV size (nm)	Standard deviation (nm)	10^th^ percentile diameter (nm)	50^th^ percentile diameter (nm)	90^th^ percentile diameter (nm)
A	1.6x10^14^	186	108	20.1	154.5	346.6
B	2.0x10^14^	213	115	109.1	214.5	408.9
C	6.1x10^14^	171	94	19.2	142.5	299.1
D	1.8x10^15^	168	104	32.2	160	332.1
Mean	6.9x10^14^	184.5	105.3	45.2	167.9	346.7

Four randomly selected milk samples underwent further characterizations of EVs (A-D). EV, extracellular vesicle; mL, milliliter; nm, nanometer.

### 3.3 EV-miRNA expression

We detected 100 EV-miRNAs that were present in every sample. EV-miRNAs present in at least 70% of participants were included in this analysis (**Supplemental Figure 4**). After filtering, there were 171,862,848 reads, with an average of 1,576,723 reads per sample. The five most highly expressed EV-miRNAs were miR-148a-3p, miR-146b-5p, miR-200a-3p, let-7g-5p, and let-7b-5p. Expression of some EV-miRNAs was highly correlated (range: -0.84, 0.96) (**Supplemental Figure 5**).

### 3.4 EV-miRNA expression profiles were associated with sequencing quality indicators, breast milk collection time, and breastfeeding

PC1 explained 18.7% of the variance in EV-miRNA CPM, and PC2 explained 15.7%. The top five loading factors contributing to PC1 were miR-148b-3p, miR-30e-5p, miR-30a-5p, miR-141-3p, and miR-30b-5p. The top five loading factors contributing to PC2 were miR-21-5p, miR-500a-3p, miR-146a-5p, miR-140-3p, and miR-629-5p.

PC analysis was used to determine whether date of EV extraction, proportion of rRNA reads, volume of skim milk, proportion of unmapped reads, and low transcriptome:genome ratio were associated with overall EV-miRNA profile (**Table 3**). We found that PC1 was associated with volume of skim milk (P_FDR_=0.004), PC2 was associated with proportion of rRNA reads (P_FDR_=3.1x10^-6)^, PC1 and 2 were associated with proportion of unmapped reads (P_FDR_=2.2x10^-11^ and 0.001, respectively), and PC1 was associated with low transcriptome:genome ratio. We did not find any technical or sequencing quality indicators that were associated with PC3-5 after FDR adjustment.

**Table 3:** Individual associations between EV-miRNA principal components and technical variables including lab variables and sequencing quality indicators.

	PC1			PC2		
	β (95% CI)	P	P_FDR_	β (95% CI)	P	P_FDR_
Lab Variables						
Date of EV extraction	1.97 (-2.68, 6.61)	0.40	0.72	-0.77 (-4.99, 3.46)	0.72	0.84
Volume of skim milk	-0.02 (-0.04, -0.009)	**0.001**	**0.004**	0.007 (-0.005, 0.02)	0.26	0.79
Sequencing Quality Indicators						
Proportion of rRNA reads	-10.75 (-33.96, 12.46)	0.36	0.72	-50.79 (-69.79, -31.80)	**6.2x10^-7^**	**3.1x10^-6^**
Proportion of unmapped reads	33.65 (25.13, 42.16)	**3.6x10^-12^**	**2.2x10^-11^**	17.34 (8.14, 26.54)	**0.0003**	**0.001**
Low transcriptome:genome ratio (Ref = No)	7.71 (3.38, 12.05)	**0.0006**	**0.003**	1.70 (-2.48, 5.89)	0.42	0.84

Beta coefficients and 95% confidence intervals (CI) from univariable linear regression analysis used to examine the associations between principal components 1 and 2 (PC1 and PC2) and technical variables including lab variables and sequencing quality indicators. P-values in bold denote statistical significance for P or P_FDR_ < 0.05. Skim milk refers to the volume of milk remaining following centrifuge.

PC analysis was used to determine whether EV-miRNA expression was associated with maternal-infant characteristics (**Table 4**). Breast milk collection time was significantly associated with PC1 (P=0.006). Predominantly breastfeeding and breastfeeding frequency were significantly associated with PC2 (P_FDR_ALL_=0.003). To determine the relative importance of each of these factors, we ran a fully adjusted model. From this analysis, we found that breast milk collection time (P=0.02) and breastfeedings/day (P=0.02) remained significantly associated with PCs 1 and 2. We examined the relationships between PC3-PC5 with maternal-infant characteristics as a sensitivity analysis (**Supplemental Table 3**). PC1-PC5 explained 48.6% of the variation in EV-miRNA expression, cumulatively. Results were largely unchanged when adjusting for proportion of unmapped reads instead of proportion of rRNA (**Supplemental Table 4**).

**Table 4:** Individual associations between miRNA principal components and maternal and infant characteristics, milk collection timing, and infant feeding characteristics.

	PC1			PC2		
	β (95% CI)	P	P_FDR_	β (95% CI)	P	P_FDR_
Maternal and infant characteristics						
Maternal age (years)	0.19 (-0.01, 0.40)	0.06	0.29	0.03 (-0.14, 0.21)	0.72	0.83
Socioeconomic status	0.02 (-0.08, 0.11)	0.71	0.83	-0.02 (-0.1, 0.06)	0.60	0.83
Pre-pregnancy BMI (kg/m^2^)	-0.06 (-0.27, 0.15)	0.60	0.83	-0.10 (-0.28, 0.08)	0.28	0.71
Maternal BMI (kg/m^2^)	-0.15 (-0.39, 0.10)	0.23	0.54	-0.09 (-0.30, 0.12)	0.40	0.71
Infant sex (ref = female)	-1.72 (-4.03, 0.60)	0.14	0.40	-0.44 (-2.42, 1.54)	0.66	0.83
Mode of delivery (ref = vaginal)	0.22 (-2.48, 2.91)	0.87	0.94	1.22 (-1.05, 3.49)	0.29	0.71
Days postpartum	0.14 (-0.20, 0.49)	0.42	0.65	-0.13 (-0.42, 0.16)	0.38	0.71
Gestational age						
Early (38-40 weeks)	-1.24 (-4.08, 1.60)	0.39	0.65	-0.69 (-3.11, 1.72)	0.57	0.83
On time (40-42 weeks)	Ref.	Ref.	Ref.	Ref	Ref.	Ref.
Late (> 42 weeks)	0.05 (-2.79, 2.89)	0.97	0.97	-0.21 (-2.63, 2.20)	0.86	0.93
Gestational diabetes (ref = no)	3.98 (-0.72, 8.67)	0.10	0.34	-1.70 (-5.72, 2.32)	0.40	0.71
Breast milk collection timing						
Season (ref = cold)	1.28 (-1.04, 3.60)	0.28	0.55	-0.03 (-2.01, 1.95)	0.98	0.98
Breast milk collection time (hours past midnight)	-1.07 (-1.82, -0.33)	**0.005**	0.07	-0.30 (-0.95, 0.36)	0.37	0.71
Infant feeding characteristics						
Predominantly breastfed	-0.45 (-2.84, 1.93)	0.71	0.83	-3.67 (-5.55, -1.77)	**0.0002**	**0.003**
Breastfeedings per day	-0.58 (-1.07, -0.09)	**0.02**	0.15	0.73 (0.33, 1.13)	**0.0005**	**0.003**

Beta coefficients and 95% confidence intervals (CI) from multivariable linear regression analysis used to examine the associations between principal components 1 and 2 (PC1 and PC2) and maternal and infant characteristics, milk collection timing, and infant feeding characteristics separately while adjusting for technical variables, including proportion of rRNA and volume of skim milk. P-values in bold denote statistical significance for P or P_FDR_ < 0.05.

### 3.5 EV-miRNA expression was associated with maternal and infant characteristics

**Figure 3:**
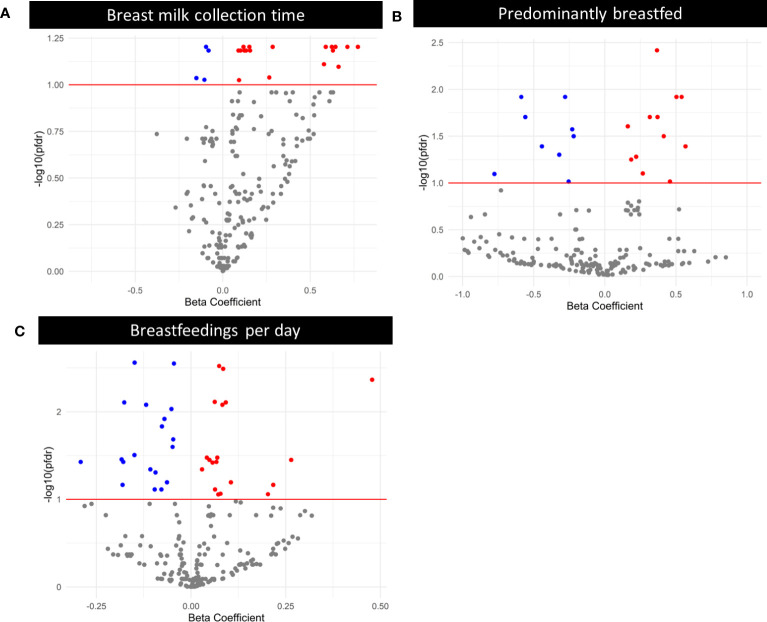
Volcano plots where each point represents the beta-estimate on the x-axis and -log_10_(P_FDR_) on the y-axis for an individual miRNA, obtained via negative binomial modeling with an offset of log(normalized library counts), and additionally adjusted for proportion of rRNA and volume of skim milk. The red line indicates the threshold for significance after Benjamini-Hochberg correction for multiple testing. Red points are statistically significant after multiple testing correction, and were positively associated with days postpartum, breast milk collection time, predominant breastfeeding, or breastfeedings per day. Blue points are statistically significant after multiple testing correction, and were negatively associated with days postpartum, breast milk collection time, predominant breastfeeding, or breastfeedings per day. **(A)** Volcano plot displaying the relationship between breast milk collection time and EV-miRNA expression. **(B)** Volcano plot displaying the relationship between predominant breastfeeding (Ref. = Yes) and EV-miRNA expression. **(C)** Volcano plot displaying the relationship between breastfeedings per day and EV-miRNA expression.

Using linear models, we examined whether expression of individual EV-miRNAs was associated with maternal and infant characteristics, adjusting for technical covariates. We found 2 EV-miRNAs that varied with number of days postpartum (miR-378e and miR-27b-3p), 23 EV-miRNAs whose expression varied based on breast milk collection time (**Figure 3A**), 23 EV-miRNAs which varied based on predominant breastfeeding status and 38 EV-miRNAs that differed by breastfeeding frequency (**Figures 3B, C**). Complete results are in **Supplemental Table 5**. As a sensitivity analysis, we also ran models adjusting for proportion of unmapped reads rather than proportion of rRNA. The associations between EV-miRNA expression and days postpartum were unchanged. There were 25 EV-miRNAs significantly associated with predominant breastfeeding and 60 associated with breastfeedings per day. However, we found that 98% of the miRNAs identified in the main analysis (adjusting for proportion of rRNA) were also identified when adjusting for proportion of unmapped reads (**Supplemental Table 6**). There were no EV-miRNAs associated with breast milk collection time when adjusting for proportion of unmapped reads. However, breast milk collection time and proportion of unmapped reads were correlated (r = -0.23, P = 0.02).

### 3.6 EV-miRNA pathway and ontology analysis

Pathway analysis of mRNA targets of miRNA associated with characteristics of interest was performed using KEGG (**Supplemental Table 7**). Here, we present only KEGG pathways that were identified by both Tarbase and microT-CDS. We identified eight KEGG pathways among EV-miRNAs that were associated with days postpartum including Hippo signaling pathway, Focal adhesion, Hippo signaling pathway, and Proteoglycans in cancer. We also identified twelve KEGG pathways among the EV-miRNAs that were associated with breast milk collection time, including: ECM-receptor interaction, Lysine degradation, and Hippo signaling pathway. For EV-miRNAs that were associated with predominant breastfeeding, ECM-receptor interaction, Proteoglycans in cancer, Pathways in cancer, and Hippo signaling pathway were among the pathways identified. For EV-miRNAs associated with breastfeedings/day, KEGG pathways identified included ECM-receptor interaction, Lysine degradation, Hippo signaling pathway, and Adherens junction, among others.

### 3.7 Proportion of unmapped reads was associated with BMI and breast milk collection time

As a secondary aim, we sought to determine whether maternal or infant characteristics were associated with the proportion of unmapped reads or rRNA. As shown in **Table 5**, we found that the proportion of unmapped reads was associated with BMI and breast milk collection time. For example, increased pre-pregnancy BMI (β=-0.004 [95% CI: -0.008, -0.003]) and BMI at 1-month postpartum (β =-0.004 [95% CI: -0.009, -0.00002]) were associated with a lower proportion of unmapped reads. Additionally, later breast milk collection time (β=-0.02 [95% CI: -0.03, -0.003]) was associated with a lower proportion of unmapped reads. Given these findings, we reexamined the associations between EV-miRNAs and pre-pregnancy BMI, BMI at 1-month postpartum, and breast milk collection time of day without adjustment for sequencing quality. Overall, we found that pre-pregnancy BMI and BMI at 1-month postpartum were not significantly associated with any EV-miRNAs when we did not adjust for proportion of rRNA. While 23 EV-miRNAs were associated with breast milk collection time when adjusting for proportion of rRNA, 14 EV-miRNAs were associated with breast milk collection time when this covariate was removed from the analysis.

**Table 5:** Univariate linear associations between sequencing quality indicators and maternal and infant characteristics, milk collection timing, and infant feeding characteristics.

	Proportion of rRNA		Proportion unmapped reads	
	β (95% CI)	P	β (95% CI)	P
Maternal and infant characteristics				
Mother age (years)	-0.0003 (-0.002, 0.001)	0.72	0.004 (-0.0002, 0.007)	0.06
Socioeconomic status	-0.0004 (-0.001, 0.0004)	0.31	0.0001 (-0.002, 0.002)	0.88
Pre-pregnancy BMI (kg/m^2^)	0.0008 (-0.001, 0.003)	0.42	-0.004 (-0.008, -0.003	**0.04**
Maternal BMI (kg/m^2^)	0.0003 (-0.002, 0.002)	0.78	-0.004 (-0.009, -0.00002)	**0.049**
Infant sex	0.02 (-0.003, 0.04)	0.10	-0.02 (-0.07, 0.02)	0.30
Mode of delivery	0.003 (-0.02, 0.03)	0.77	-0.02 (-0.07, 0.03)	0.46
Days postpartum	0.0003 (-0.003, 0.003)	0.82	-0.0003 (-0.007, 0.006)	0.92
Gestational age				
Breast milk collection timing				
Season (ref = cold)	0.01 (-0.006, 0.03)	0.16	-0.01 (-0.05, 0.03)	0.65
Breast milk collection time (hours past midnight)	-0.003 (-0.01, 0.003)	0.30	-0.02 (-0.03, -0.003)	**0.02**
Infant feeding characteristics				
Predominantly breastfed	-0.003 (-0.02, 0.02)	0.73	0.02 (-0.02, 0.01)	0.27
Breastfeedings per day	-0.002 (-0.006, 0.002)	0.29	0.002 (-0.007, 0.01)	0.67

Beta coefficients and 95% confidence intervals (CI) from univariate linear regression analysis used to examine the associations between sequencing quality indicators and maternal and infant characteristics, milk collection timing, and infant feeding characteristics. P-values in bold denote statistical significance for P or P_FDR_ < 0.05.

## 4 Discussion

This study systematically examined technical and maternal-infant characteristics that may impact expression of breast milk EV-miRNAs. We found that differences in EV-miRNA expression, as captured by PC analysis, were associated with predominant breastfeeding and breastfeeding frequency. Further, we identified individual EV-miRNAs that were associated with days postpartum, breast milk collection time, predominant breastfeeding, and breastfeeding frequency. These findings indicate that maternal-infant characteristics should be considered in human studies of milk EV-miRNAs.

Our study was similar to other previous studies in several ways. First, in characterizing EV isolation, we determined that we were able to isolate EVs which were of the expected size and similar to other comparable studies (15, 17). However, we did isolate a higher concentration of EV particles in comparison with other, similar studies (15). Milk EV concentrations and sizes can vary greatly by milk processing and EV isolation methods, which likely have resulted in the observed differences between studies. However, several studies have observed EVs in milk with similar average size ranges (30-300 nm) (15, 17, 32, 33). Differences in EV concentrations may be due to some contamination by casein micelles, which may co-isolate with EVs, or to differences in lactation stage, which may influence EV levels. Next, we identified several EV-miRNAs that differed based on days postpartum, which is similar to findings from a previous study (15). In contrast to prior work, we did not find that EV-miRNA expression was associated with infant sex (34). We did not find that pre-pregnancy BMI was associated with EV-miRNA expression. This differs from previous studies, which have identified that pre-pregnancy BMI was inversely associated with the expression of several miRNAs, including hsa-miR-148a, hsa-miR-30b, let-7a, and hsa-miR-378 (15, 16, 34). This difference may be due to variations in milk collection time (i.e., colostrum vs. mature milk) or the study population examined since participants in the current study largely had overweight or obesity. In addition to identifying several EV-miRNAs that were associated with breastfeeding, we also found several KEGG pathways associated with predominant breastfeeding, and/or breastfeeding frequency. For instance, Hippo signaling pathway and ECM-receptor interaction were identified across multiple characteristics of interest. Hippo signaling pathway is a pathway that controls organ size in humans, while the ECM-interaction pathway plays an important role in tissue and organ morphogenesis and maintaining the structure and function of cells and tissues (31).

As a secondary analysis, we examined whether maternal and/or infant characteristics predicted the proportion of unmapped reads or rRNA. Overall, we observed that both maternal BMI and breast milk collection time were inversely associated with the proportion of unmapped reads, suggesting that lower BMI and later breast milk collection time may be related to increased RNA editing or increased presence of exogenous genomes. Based on this, we hypothesized that the proportion of unmapped reads may mediate some of the observed associations between breast milk EV-miRNA expression with breast milk collection time. Indeed, we identified several differences in the EV-miRNAs that were associated with breast milk collection time depending on whether we adjusted for the proportion of unmapped reads in our analysis. These findings suggest that future studies should consider standardizing or otherwise empirically adjusting for time of breast milk collection.

A primary strength of this study is the assessment of breast milk EV-miRNAs in the context of several detailed measures of maternal and infant characteristics during the early postpartum period. Despite this, there are weaknesses that are worth noting. First, as the primary aim of this study was not EV analysis, human milk samples were frozen, which may have resulted in contamination by intracellular vesicles from lysed cells or a substantial loss of milk EVs (35). However, prior work has shown that EV isolation from frozen milk is feasible (36) and several prior studies have successfully isolated EVs from previously frozen milk samples (15–17). Furthermore, a representative subset of human milk EV samples was positive for eight EV markers and had higher levels of these markers compared to their EV-depleted counterparts, suggesting that we enriched our samples for EVs over other biological materials. Our samples showed minimal contamination by GM130, a Golgi matrix protein that can serve as a marker for cellular contamination. While analyses of frozen breast milk samples may not be comparable to fresh milk samples, it is common practice for milk to be refrigerated or frozen prior to infant feeding, so it is important to understand the biological and technical factors impacting human milk in both contexts.

Further, previous studies have found that the methods we used for EV isolation may have low specificity for EVs, obtaining larger particles as well (37). However, we employed differential centrifugation to remove larger particles prior to EV isolation and nanoparticle tracking analysis determined that the mean EV size was 184.5 nm in a randomly chosen subset of human milk EV samples, without contaminating larger particles and TEM visualization confirmed that the isolated EVs were of the expected size. Another limitation of our study was the inability to quantify casein and whey contamination in our samples. Casein and whey are the major milk proteins. Although EVs are present in the whey fraction of milk, due to the ability of casein to form micelles, it can contaminate EV preparations based on size-affinity (33). Numerous studies have examined casein contamination in bovine milk, where it comprises up to 80% of milk proteins (33, 38, 39). However, casein comprises approximately 35% of human milk proteins (40). To date, few human milk studies have examined the associations of casein and whey levels with EV miRNA expression. In a previous study, caseins were not detected in human milk EVs isolated with ultracentrifugation (41). However, other studies found beta-casein present in milk EV fractions (36). Although we did not observe large amounts of protein contamination in our TEM images (See **Figure 2**), casein and whey may still be present in our EV fraction. Nonetheless, this contamination is only relevant to our study should it influence miRNA expression levels. Previous studies in bovine milk found that there was no difference in miRNA expression between EVs acidified to remove casein and untreated EVs (42). More work is necessary in this field. Follow-up analyses would benefit from analysis of miRNA expression in human milk EVs in relation to casein and whey levels.

Additionally, we cannot disentangle breastfeeding frequency and time since last breastfeeding. Breast milk samples were collected > 1.5 hours after the last feeding, but participants were not asked when they had last breastfed. Thus, breastfeedings/day may be a proxy for time since last breastfeeding. Future studies should ascertain time since last feeding at the time of breast milk collection. Our study population consisted of Latino women who had overweight or obesity, which may limit the generalizability of our findings. This cross-sectional analysis was also conducted in the early postpartum period where infants were between 23 and 46 days of age, which limited our ability to assess the full course of lactation. However, one previous study examining human milk exosomal miRNA found that differences in miRNA expression by maternal BMI present at one-month did not persist to three-months of infant age, suggesting that the first month may be a critical period (16). Further analysis is needed to characterize important factors in breast milk EV-miRNA over the course of lactation. Finally, gestational diabetes has previously been associated with EV-miRNA expression (18), but we were unable to assess whether breast milk EV-miRNA expression differed by diabetes status in this cohort due to low prevalence of gestational diabetes in our population.

In conclusion, this study expands on our previous understanding of EV-miRNA expression by identifying technical and participant factors associated with EV-miRNA expression in Latino mother-child pairs. Our findings indicate that future analyses should consider breast milk collection time, predominant breastfeeding, and breastfeeding frequency as potentially important covariates. We anticipate that results from this study will inform future analyses focused on breast milk EV-miRNAs.

## Data availability statement

The datasets presented in this article are not readily available because of ethical and privacy restrictions, as the data contains potentially identifiable participant information. Requests to access the datasets should be directed to Tanya.Alderete@colorado.edu.

## Ethics statement

The studies involving human participants were reviewed and approved by The Institutional Review Boards of the University of Southern California, Children’s Hospital of Los Angeles, and the University of Colorado Boulder. Written informed consent to participate in this study was provided by the participants’ legal guardian/next of kin.

## Author contributions

EH conducted the formal analyses and drafted the article. AK performed laboratory analysis and QC of miRNA sequencing results. TA, AK, and MG conceived of the study design and supervised formal analyses. MG developed the cohort and oversaw sample collection. WP, BC, and TA reviewed code and confirmed the outputs included in the manuscript. All authors assisted in the interpretation of findings, critically revised the article, and approved the version to be published.
