# Supplementary material for: Influence of technical and maternal-infant factors on the measurement and expression of extracellular miRNA in human milk
Source: Front Immunol. 2023 Jul 10;14:1151870. doi: 10.3389/fimmu.2023.1151870 (PMC10363855; doi:10.3389/fimmu.2023.1151870)
Supplement: Supplementary file 1 [file DataSheet_1.docx]

Supplementary Material

Influence of technical and maternal-infant factors on the measurement and expression of extracellular miRNA in human milk

Elizabeth A Holzhausen, Allison Kupsco, Bridget Chalifour, William B Patterson, Kelsey A Schmidt, Pari Mokhtari, Andrea A Baccarelli, Michael I Goran, Tanya L Alderete^*^

*** Correspondence:** Tanya L Alderete: Tanya.Alderete@colorado.edu

# Supplementary Figures and Tables

## Supplementary Figures





**Supplementary Figure 1.** Plot enumerating the proportion of variance explained by principal components 1 through 10.

**Supplemental Figure 2.** Directed Acyclic Graphs Illustrating Sequencing Quality Indicators may Mediate the Relationships between Maternal BMI and Breast Milk Collection Time with EV-miRNA Expression.


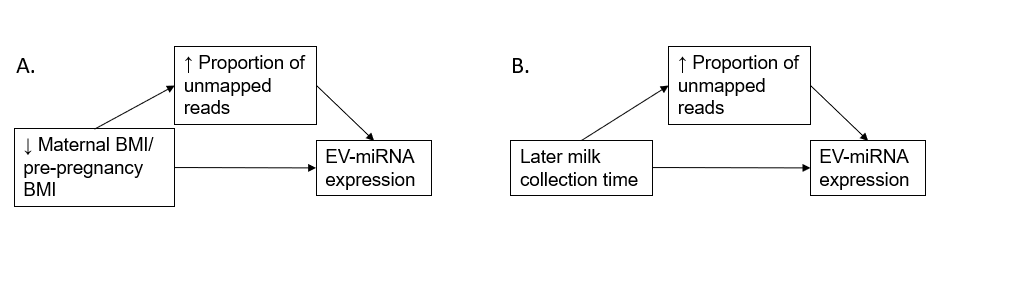
**Supplemental Figure 2.** Figures illustrating the proposed relationships between (A) maternal and pre-pregnancy BMI, proportion of unmapped reads, and EV-miRNA expression, and (B) the proposed relationships between milk collection time, proportion of unmapped reads, and EV-miRNA expression.

**Supplemental Figure 3.** Results from Exo-Check Array demonstrating exosome-related protein expression in pooled samples of three human milk EV samples (left) and two of the corresponding samples EV-depleted samples (right).


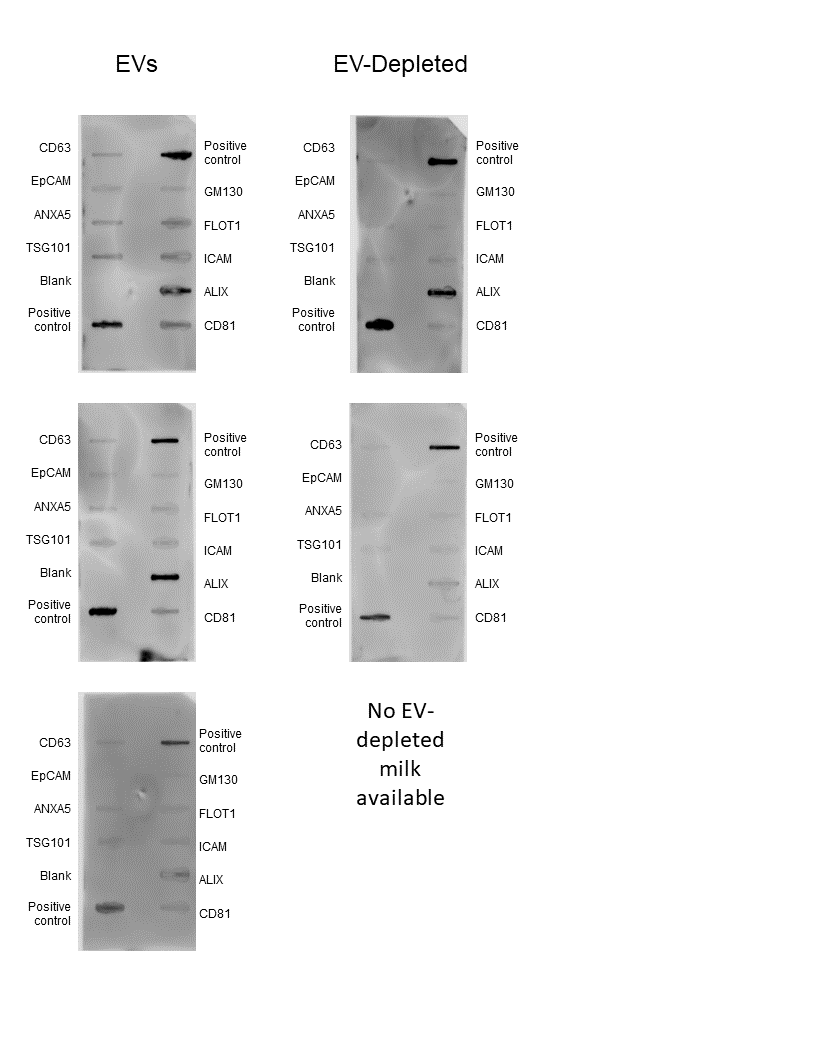


**Supplemental Figure 3.** Darkness of each line indicates the presence of the indicated protein. Abbreviations: GM130: Cis-golgi matrix protein, FLOT1: Flotillin-1, ICAM1: Intracellular adhesion molecule 1, ALIX: Programmed cell death 6 interacting protein (PDCD6IP), CD81: Tetraspanin, CD63: Tetraspanin, EpCam: Epithelial cell adhesion molecule, ANXA5: Annexin A5, TSG101: Tumor susceptibility gene 101.

**Supplemental Figure 4.** Plot showing the number of unique miRNAs remaining in our sample after applying varying thresholds for removal. E.g., if we require that miRNAs be present in at least 10% of samples, we retain 488 unique miRNAs in our analysis.





**Supplemental Figure 5.** Spearman’s correlations between the 210 EV-miRNAs in our analysis, sorted using hierarchical clustering.


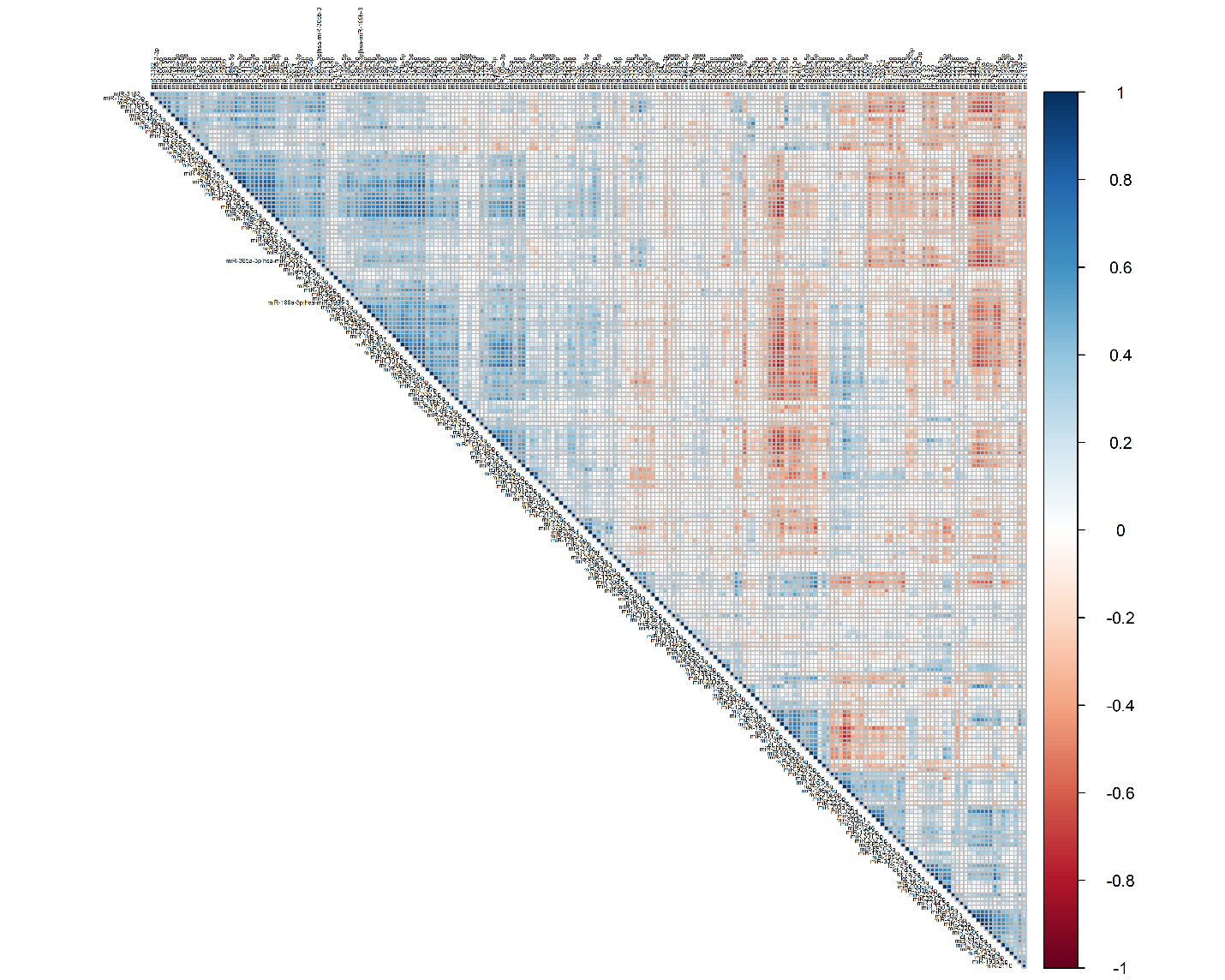


## Supplemental Tables

**Supplemental Table 1.** Baseline characteristics of 209 Hispanic mother-infant dyads from the Southern California Mother’s Milk Study.

| **Maternal and infant characteristics** | **Mean (SD) or N, %**  **Excluded from miRNA analysis**  **(n = 100)** | **Mean (SD) or N, %**  **Subset with miRNA data**  **(n = 109)** | **P-value** |
| --- | --- | --- | --- |
| Maternal age (years) | 30.0 ± 6.5 | 28.0 ± 5.6 | **0.02** |
| Socioeconomic status | 27.3 ± 12.2 | 25.6 ± 12.2 | 0.3 |
| Pre-pregnancy BMI (kg/m^2^) | 30.7 ± 5.7 | 28.3 ± 5.4 | 0.5 |
| Maternal BMI (kg/m^2^) |  | 29.9 ± 4.7 | 0.3 |
| Normal weight (BMI < 25) |  |  |  |
| Overweight (25 ≤ BMI < 30) |  |  |  |
| Obese (BMI ≥ 30) |  |  |  |
| Infant sex (female, male, % female) | 52, 48, 52% | 60, 49, 55% | 0.8 |
| Mode of delivery (vaginal, caesarean, % vaginal) | 74, 26, 74% | 83, 26, 76% | 0.8 |
| Days postpartum | 32.7 ± 5.6 | 32.5 ± 3.3 | 0.7 |
| Gestational age  Early (38-40 weeks gestation)  On time (40 weeks gestation)  Late (40-42 weeks gestation) | 21, 21%  57, 57%  22, 22% | 27, 25%  55, 50%  27, 25% | 0.6 |
| Gestational Diabetes^a^ (Yes, No, %Yes) | 5, 94, 5.1% | 7, 102, 6.4% | 0.9 |
| **Breast milk collection timing** | **Mean (SD) or N, %**  **Excluded from miRNA analysis** | **Mean (SD) or N, %**  **Subset with miRNA data** | **P-value** |
| Season (cold, warm, % cold) | 43, 57, 43% | 59, 50, 54% | 0.1 |
| Breast milk collection time | 11.4 ± 1.4 | 11.3 ± 1.5 | 0.7 |
| **Infant feeding characteristics** | **Mean (SD) or N, %**  **Excluded from miRNA analysis** | **Mean (SD) or N, %**  **Subset with miRNA data** | **P-value** |
| Predominantly breastfed (yes, no, % yes) | 45, 55, 55% | 48, 61, 44% | 0.1 |
| Breast feedings per day | 7.1 ± 1.9 | 6.4 ± 2.4 | **0.02** |

**Supplemental Table 1.** Baseline (1-month) characteristics of 209 Hispanic mother-infant dyads from the Southern California Mother’s Milk Study. For continuous variables, independent t-tests were used to test for differences between the full sample, and the subset of the sample for whom breast milk miRNAs were measured. For categorical variables, chi-square tests were used to test for differences between the full sample and the subset of the sample for whom breast milk miRNAs were measured. ^a^Among observations excluded from miRNA analysis, the overall N for gestational diabetes was 99; one participant was missing gestational diabetes information.

**Supplemental Table 3.** Individual associations (adjusted for technical covariates) between miRNA principal components 3-5 and maternal and infant characteristics, milk collection timing, and infant feeding characteristics.

|  | **PC3** | | | **PC4** | | | **PC5** | | |
| --- | --- | --- | --- | --- | --- | --- | --- | --- | --- |
|  | **β (95% CI)** | **p** | **p_fdr_** | **β (95% CI)** | **p** | **p_fdr_** | **β (95% CI)** | **p** | **p_fdr_** |
| Maternal and infant characteristics | | | | | | | | | |
| Maternal age (years) | 0.05 (-0.07, 0.17) | 0.43 | 0.62 | 0.03 (-0.08, 0.14) | 0.56 | 0.91 | 0.03 (-0.08, 0.14) | 0.56 | 0.91 |
| Socioeconomic status | 0.01 (-0.05, 0.07) | 0.72 | 0.85 | 0.03 (-0.02, 0.08) | 0.25 | 0.66 | 0.03 (-0.02, 0.08) | 0.25 | 0.66 |
| Pre-pregnancy BMI (kg/m^2^) | -0.03 (-0.15, 0.10) | 0.66 | 0.85 | 0.07 (-0.04, 0.19) | 0.21 | 0.66 | -0.01 (-0.11, 0.08) | 0.81 | 0.96 |
| Maternal BMI (kg/m^2^) | -0.08 (-0.23, 0.06) | 0.25 | 0.40 | 0.03 (-0.10, 0.16) | 0.91 | 0.91 | 0.03 (-0.10, 0.16) | 0.64 | 0.91 |
| Infant sex  (ref = female) | 0.13 (-1.24, 1.49) | 0.86 | 0.86 | 0.07 (-1.18, 1.32) | 0.91 | 0.91 | 0.07 (-1.18, 1.32) | 0.25 | 0.66 |
| Mode of delivery  (ref = vaginal) | -1.74 (-3.27, -0.21) | **0.03** | 0.32 | 0.08 (-1.36, 1.53) | 0.91 | 0.91 | 0.08 (-1.36, 1.53) | 0.91 | 0.91 |
| Days post-partum | 0.12 (-0.08, 0.32) | 0.25 | 0.40 | -0.12 (-0.31, 0.06) | 0.18 | 0.66 | -0.12 (-0.31, 0.06) | 0.18 | 0.66 |
| Gestational age |  |  |  |  |  |  |  | | |
| Early (38-40 weeks) | -1.46 (-3.09, 0.17) | 0.08 | 0.32 | -0.48 (-2.00, 1.05) | 0.54 | 0.91 | -0.48 (-2.00, 1.05) | 0.54 | 0.91 |
| On time (40-42 weeks) | Ref. | Ref. | Ref. | Ref. | Ref. | Ref. | Ref. | Ref. | Ref. |
| Late (> 42 weeks) | -1.45 (-3.08, 0.18) | 0.08 | 0.32 | -0.12 (-1.64, 1.41) | 0.88 | 0.91 | -0.12 (-1.64, 1.41) | 0.88 | 0.91 |
| Milk collection timing | | | | | | | | | |
| Season (ref = cold) | -0.99 (-2.33, 0.36) | 0.15 | 0.39 | -1.18 (-2.41, 0.05) | 0.06 | 0.48 | -1.18 (-2.41, 0.05) | 0.06 | 0.48 |
| Breast milk collection time (hrs. past midnight) | 0.04 (-0.40, 0.49) | 0.84 | 0.86 | 0.37 (-0.04, 0.78) | 0.07 | 0.48 | 0.37 (-0.04, 0.19) | 0.21 | 0.66 |
| Infant feeding characteristics | | | | | | | | | |
| Predominantly breast fed | 1.15 (-0.22, 0.06) | 0.25 | 0.40 | -0.09 (-1.37, 1.19) | 0.89 | 0.91 | -0.09 (-1.37, 1.19) | 0.89 | 0.91 |
| Breast feedings per day | -0.17 (-0.46, 0.12) | 0.24 | 0.40 | -0.02 (-0.03, 0.25) | 0.91 | 0.91 | -0.02 (-0.29, 0.25) | 0.91 | 0.91 |
| Gestational diabetes  (ref = No) | -0.44 (-3.21, 2.33) | 0.75 | 0.86 | 1.47 (-1.06, 4.01) | 0.25 | 0.59 | 0.33 (-1.80, 2.45) | 0.76 | 0.94 |

Supplemental Table 3. Beta coefficients and 95% confidence intervals (CI) from multivariable linear regression analysis used to examine the associations between principal components 3-5 and maternal and infant characteristics, milk collection timing, and infant feeding characteristics separately while adjusting for technical variables, including proportion of rRNA and volume of skim milk. *P*-values in bold denote statistical significance for *p* or *p*_fdr_ < 0.05.

**Supplemental Table 4.** Individual associations between miRNA principal components and maternal and infant characteristics, milk collection timing, and infant feeding characteristics.

|  | **PC1** | | | **PC2** | | |
| --- | --- | --- | --- | --- | --- | --- |
|  | **β (95% CI)** | **p** | **p_fdr_** | **β (95% CI)** | **p** | **p_fdr_** |
| Maternal and infant characteristics | | | | | | |
| Maternal age (years) | 0.07 (-0.09, 0.23) | 0.36 | 0.59 | -0.01 (-0.20, 0.18) | 0.90 | 1.00 |
| Socioeconomic status | 0.02 (-0.05, 0.09) | 0.62 | 0.73 | -9.8x10^-6^ (-0.09, 0.09) | 1.00 | 1.00 |
| Pre-pregnancy BMI (kg/m^2^) | 0.08 (-0.08, 0.25) | 0.33 | 0.59 | -0.75 (-0.27, 0.12) | 0.45 | 0.97 |
| Maternal BMI (kg/m^2^) | 0.0007 (-0.19, 0.19) | 0.99 | 0.99 | -0.03 (-0.26, 0.19) | 0.76 | 1.00 |
| Infant sex  (ref = female) | -1.03 (-2.78, 0.72) | 0.24 | 0.59 | -0.90 (-2.98, 1.19) | 0.39 | 0.97 |
| Mode of delivery  (ref = vaginal) | 0.84 (-1.20, 0.29) | 0.42 | 0.60 | 1.36 (-1.06, 3.78) | 0.27 | 0.97 |
| Days post-partum | 0.14 (-0.12, 0.41) | 0.27 | 0.59 | -0.15 (-0.46, 0.16) | 0.35 | 0.97 |
| Gestational age |  |  |  |  |  |  |
| Early | -0.80 (-2.94, 1.35) | 0.46 | 0.60 | 0.15 (-2.42, 2.71) | 0.91 | 1.00 |
| On time | Ref. | Ref. | Ref. | Ref | Ref. | Ref. |
| Late | -1.03 (-3.18, 1.13) | 0.34 | 0.59 | 0.05 (-2.52, 2.63) | 0.97 | 1.00 |
| Milk collection timing | | | | | | |
| Season (ref = cold) | 1.54 (-0.20, 3.27) | 0.08 | 0.51 | -0.53 (-2.62, 1.56) | 0.61 | 1.00 |
| Breast milk collection time (hours past midnight) | -0.47 (-1.06, 0.12) | 0.12 | 0.51 | 0.17 (-0.54, 0.88) | 0.63 | 1.00 |
| Infant feeding characteristics | | | | | | |
| Predominantly breastfed | 0.28 (-1.54, 2.10) | 0.76 | 0.83 | -3.58 (-5.63, -1.54) | **0.0008** | **0.005** |
| Breastfeedings per day | -0.58 (-0.95, -0.22) | **0.002** | **0.02** | 0.85 (0.43, 1.27) | **0.0001** | **0.001** |

**Supplemental Table 4.** Beta coefficients and 95% confidence intervals (CI) from multivariable linear regression analysis used to examine the associations between principal components 1 and 2 (PC1 and PC2) and maternal and infant characteristics, milk collection timing, and infant feeding characteristics separately while adjusting for technical variables, including proportion of unmapped reads and volume of skim milk. *P*-values in bold denote statistical significance for *p* or *p*_fdr_ < 0.05.
